# Prolonged Fatigue and Mental Health Challenges in Critical COVID-19 Survivors

**DOI:** 10.1177/08850666241255328

**Published:** 2024-07-23

**Authors:** Malin Hultgren, Ingrid Didriksson, Anders Håkansson, Sara Andertun, Attila Frigyesi, Erik Mellerstedt, Maria Nelderup, Anna C. Nilsson, Anton Reepalu, Martin Spångfors, Hans Friberg, Gisela Lilja

**Affiliations:** 1 AT/ST, Department of Strategic Healthcare Development and Security, Skåne University Hospital, Lund, Sweden; 2 Anaesthesia and Intensive Care, 156327 Department of Clinical Sciences Lund, Lund University, Lund, Sweden; 3 Intensive and Perioperative Care, Skåne University Hospital, Malmö, Sweden; 4 Malmö Addiction Centre, Clinical Research Unit, Skåne University Hospital, Malmö, Sweden; 5 Division of Psychiatry, 156327 Department of Clinical Sciences Lund, Lund University, Lund, Sweden; 6 Anaesthesia and Intensive Care and Clinical Sciences Helsingborg, Department of Clinical Sciences Lund, Helsingborg Hospital, Lund University, Lund, Sweden; 7 Department of Intensive Care Medicine, Helsingborg Hospital, Helsingborg, Sweden; 8 Intensive and Perioperative Care, Skåne University Hospital, Lund, Sweden; 9 Department of Infectious Diseases, Skåne University Hospital, Lund, Sweden; 10 Department of Infectious Diseases, Skåne University Hospital, Malmö, Sweden; 11 Department of Translational Medicine, Lund University, Malmö, Sweden; 12 Anaesthesia and Intensive Care, Kristianstad Hospital, Kristianstad, Sweden; 13 Neurology, Skåne University Hospital, Lund, Sweden; 14 Neurology, Department of Clinical Sciences Lund, Lund University, Lund, Sweden

**Keywords:** COVID-19, critical care outcomes, critical illness, fatigue, intensive care, mental illness, patient-reported outcome measures, post-acute COVID-19 syndrome, prolonged fatigue and mental health challenges in critical COVID-19 survivors

## Abstract

**Background:** The aim of this study was to investigate the development of fatigue and mental illness between 3 and 12 months after critical COVID-19 and explore risk factors for long-lasting symptoms. **Study Design and Methods:** A prospective, multicenter COVID-19 study in southern Sweden, including adult patients (≥18 years) with rtPCR-confirmed COVID-19 requiring intensive care. Survivors were invited to a follow-up at 3 and 12 months, where patient-reported symptoms were assessed using the Modified Fatigue Impact Scale (MFIS), the Hospital Anxiety and Depression Scale (HADS) and the Posttraumatic Stress Disorder Checklist version 5 (PCL-5). The development between 3 and 12 months was described by changes in relation to statistical significance and suggested values for a minimally important difference (MID). Potential risk factors for long-lasting symptoms were analyzed by multivariable logistic regression. **Results:** At the 3-month follow-up, 262 survivors (87%) participated, 215 (72%) returned at 12 months. Fatigue was reported by 50% versus 40%, with a significant improvement at 12 months (MFIS; median 38 vs. 33, *P *< .001, MID ≥4). There were no significant differences in symptoms of mental illness between 3 and 12 months, with anxiety present in 33% versus 28%, depression in 30% versus 22%, and posttraumatic stress disorder in 17% versus 13%. A worse functional outcome and less sleep compared to before COVID-19 were risk factors for fatigue and mental illness at 12 months. **Conclusions:** Fatigue improved between 3 and 12 months but was still common. Symptoms of mental illness remained unchanged with anxiety being the most reported. A worse functional outcome and less sleep compared to before COVID-19 were identified as risk factors for reporting long-lasting symptoms.

## Introduction

Several studies have reported on long-lasting symptoms after acute coronavirus disease 2019 (COVID-19), often named post-acute COVID-19 syndrome (PACS). The prevalence of PACS varies and is common regardless of the severity of the acute disease.^1^ Individuals who have experienced a more severe COVID-19, however, are reported to have an increased risk of PACS compared to those with a less severe disease. PACS is estimated to affect nearly half of the hospitalized patients with COVID-19.^1–5^

Survivors of intensive care in general may experience new or worsening symptoms in mental health, cognitive and physical function, known as postintensive care syndrome (PICS).^6^ A common reason for intensive care is acute respiratory failure that may develop to an acute respiratory distress syndrome (ARDS), among whom 20–40% report lasting symptoms of depression, anxiety, and posttraumatic stress disorder (PTSD).^7,8^ In addition to symptoms of mental illness, survivors of ARDS often report fatigue, which has been observed in more than two-thirds during the first year, and is associated with symptoms of mental illness.^9^

There are thus similarities between PACS, PICS and post-ARDS since symptoms of fatigue and mental illness are prevalent in all these populations. The current knowledge on the dynamics of symptoms of fatigue and mental illness among survivors of critical COVID-19 is still limited.^1,8–10^ In a Dutch study, 26% of survivors of critical COVID-19 requiring intensive care experienced symptoms of mental illness after 12 months, and 56% reported fatigue.^10^ Participants were, however, only assessed once and the study lacks information regarding development over time and about potential risk factors.

In addition, overall functional outcome as assessed by the Glasgow Outcome Scale Extended (GOSE) was reported to improve between 3 and 12 months, but its association with fatigue and mental illness after critical COVID-19 has not been investigated.^11^

The aim of this study was to investigate the development of symptoms of fatigue and mental illness between 3 and 12 months after critical COVID-19 and explore risk factors for long-lasting symptoms among survivors.

## Study Design and Methods

### Design, Setting, and Participants

This multicenter, observational cohort study is part of the SWECRIT COVID-19 study, conducted in the southern healthcare region in Sweden, Region Skåne. Between May 11, 2020 and May 10, 2021, adult patients (≥18 years) admitted to any of the 6 participating intensive care units (ICU) with reverse transcription polymerase chain reaction-confirmed COVID-19 were eligible for inclusion in the main study.^11^ Participants in this follow-up study were survivors of critical COVID-19, defined as a COVID-19 infection requiring intensive care with a ratio of arterial oxygen partial pressure to the fractional inspired oxygen of ≤40 kPa. The enrollment in the population-based study was predetermined to close after 1 year; therefore, no a priori power analysis was performed. The manuscript was prepared per the STROBE guidelines for observational studies.^12^

Patients admitted to the ICU with COVID-19 during the inclusion period were recruited to the study in conjunction with admission to the ICU. Potential participants received oral and written information about the study in the ICU by physicians or research nurses. Consent was obtained by physicians or research nurses. If consent could not be obtained at admission, written consent was obtained during ICU-stay, hospital stay, or prior to a clinical follow-up. Consent was presumed for deceased patients. Survivors were invited to a face-to-face clinical follow-up visit at 3 and 12 months after admission to the ICU. As an alternative, the follow-up could be performed as a telephone interview. Outcome assessors were occupational therapists, physiotherapists, registered nurses, or physicians, who had received mandatory training to reduce interrater variability. Authorized interpreters were used when deemed necessary by the assessor or the participant. No information regarding ethnicity apart from if a participant's native language was Swedish was collected.

Participants received questionnaires and instructions via mail before the follow-up visit. The last follow-up visit was conducted in August 2022. Follow-ups were not conducted before, but as close to 3 and 12 months after admission as possible.

The SWECRIT COVID-19 study was approved by the Swedish Ethical Review Authority (2020/01955, 2020/03483, 2020/05233, 2021/00655).

### Outcomes and Outcome Measures

The primary outcome was the development of patient-reported symptoms of fatigue and mental illness between 3 and 12 months. Mental illness included symptoms of anxiety, depression, and PTSD.

The Modified Fatigue Impact Scale (MFIS)—derived from the Fatigue Impact Scale—is a patient-reported instrument used to identify and quantify the impact and type of fatigue.^13^ The MFIS has been used somewhat in populations recovering from COVID-19, and has been used extensively to evaluate fatigue in chronic illnesses like multiple sclerosis.^14–16^ The MFIS contains 21 items and utilises a 5-point ordinal scale on each item (0–4) with a total score range 0–84. It can be also be subdivided into its 3 constituent subscales to facilitate comparison of fatigue's impact on physical, cognitive, and psychosocial functioning by using a mean value for each subscale. A cutoff of ≥38 for the total score was used to determine fatigue.^17^ The minimal important difference (MID) was set to ≥4, based on estimates from a population with multiple sclerosis.^18^

Symptoms of anxiety and depression were assessed by the Hospital Anxiety and Depression Scale (HADS), which is recommended as a core outcome measure for survivors of ARDS.^19,20^ The HADS contains 14 items divided into 2 domains: HADS-Anxiety (HADS-A) and HADS-Depression (HADS-D), with 7 items for each domain. Each item is rated on an ordinal 4-point scale (0–3), adding up to a total score of 0–21 for each domain. A cutoff score of ≥8 indicates significant symptoms in the respective domain.^21^ A change of ≥2 points is proposed as a MID in survivors of acute respiratory failure.^22^

Patient-reported symptoms of PTSD were assessed by the Posttraumatic Stress Disorder Checklist updated for DSM-5 (PCL-5), which contains 20 items, using a 5-point ordinal scale on each item (0–4), with a total score range 0–80.^23–25^ The cutoff was set to ≥33.^26^ In addition, the MID was set to a change of ≥6 points, based on a study population with chronic pain and comorbid depression and/or anxiety.^27^

Both the HADS and PCL-5 have been shown to be valid instruments for the assessment of anxiety, depression, and PTSD.^20,21,25^

Data regarding patient demographics, age, sex, clinical frailty,^28^ body mass index (BMI), Charlson comorbidity index,^29,30^ and variables concerning ICU care (Simplified Acute Physiology Score 3, prone positioning, invasive ventilation, and time in mechanical ventilation) were collected from a regional quality register, COVID-IR. Further descriptive data were collected at the follow-ups, which included detailed participant characteristics (native language, living situation, working status prior/post illness, rehabilitation, changes in sleep quantity compared to before COVID-19) and data on functional outcome as assessed by the GOSE. GOSE is a clinician-reported assessment that utilizes an ordinal scale with 8 categories to report on overall functional outcome, ranging from “1” representing death to “8” representing a full recovery (upper level of good recovery).^31^

### Statistical Analysis

Descriptive statistics are presented as mean with standard deviation (SD), or median with interquartile range (IQR). For all patient-reported instruments, a higher score indicates worse symptoms. Categorical data are presented as frequencies (*n*) and percentages (%). If ≤3 items were missing in a questionnaire, subscale mean-value imputation was utilized.^32^

The paired Wilcoxon Signed rank test was used to compare scores for MFIS, HADS-A, HADS-D, and PCL-5 between 3 and 12 months. A *P* value of <.05 was considered significant. A subgroup analysis of native versus nonnative Swedish speakers comparing patient-reported symptoms was performed using the independent samples Mann–Whitney *U* test. The dynamics in the scores between 3 and 12 months were described for the whole cohort by using a minimal important difference, and categorized as less, worse, or unchanged symptoms.

To explore risk factors for long-lasting symptoms (12 months), logistic regression was used. The dependent variable was the dichotomous variable of participants with or without symptoms at 12 months (above or below cutoff), with separate models created for each symptom. The independent variables were *age, gender*; preexisting comorbidities (*hypertension, diabetes mellitus, cardiovascular disease*, *chronic pulmonary disease*), proxies for disease severity (*ICU-length of stay, Simplified Acute Physiology Score 3*, *invasive ventilation),* and at 12 months *rehabilitation, functional outcome (GOSE)*, *less sleep compared to before COVID-19 *and* more sleep compared to before COVID-19*. Functional outcome (GOSE) was included as a continuous variable in the models. For changes in sleeping quantity compared to before COVID-19 2 dichotomous variables (yes/no) of “less” or “more” sleep compared to before COVID-19 were used. Akin to “purposeful selection,” each variable was first analyzed in a univariable binary logistic regression model with a *P *value cutoff of *P* < .25.^33^ If more variables were below the cutoff in the first stage than were viable for the model (a maximum of 1 variable for every ten events per symptom), the variables with the lowest *P* values were prioritized. Relevant variables were then added to the multivariable model in a single step. A *P* value < .05 was considered significant in the final multivariable model. The Hosmer–Lemeshow test was used to evaluate the model calibration. Due to the exploratory design, no further adjustments for multiple analyses were performed and all results were considered hypothesis-generating only. Statistical analyses were performed using IBM Corporation SPSS Statistics for Mac, Version 27.0, released in 2020, Armonk, NY, USA.

## Results

**Figure 1.**
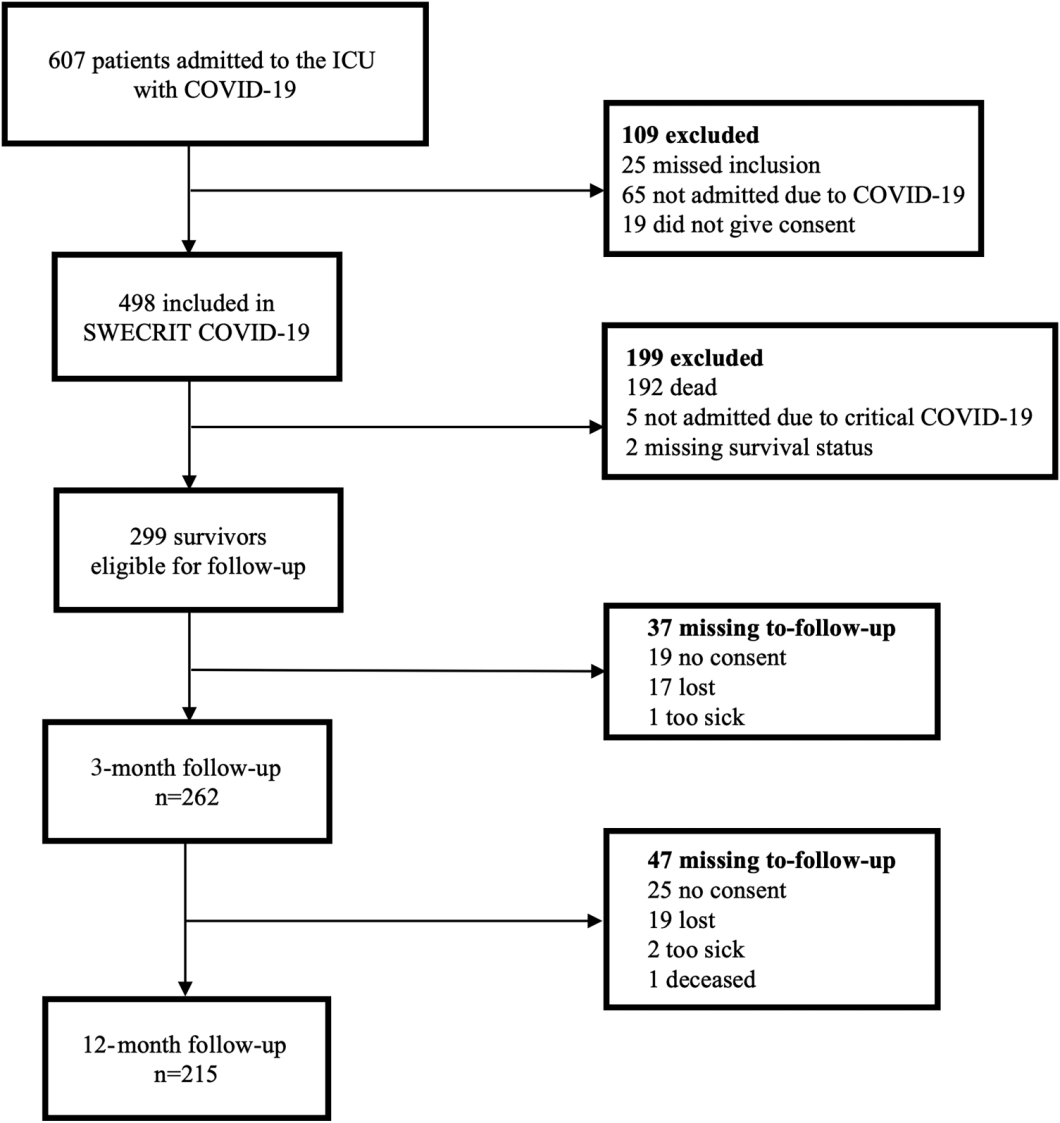
Flowchart of inclusion and reasons for exclusion/missing to-follow-up. The study's inclusion period was between May 11, 2020 and May 10, 2021.

At 3 months, 299 of 498 (60%) participants were alive and eligible for inclusion (Figure 1). Of these, 262/299 (87%) participated in the 3-month follow-up, and 215/298 (72%) participated at 12 months. The majority of follow-ups were conducted through face-to-face interviews at 3 (173/262, 66%) and 12 months (158/215, 73%).

Participants had a median age of 61 (52–68) years, and 74% were male (Table 1). Common comorbidities were hypertension (51%) and diabetes mellitus (29%). Baseline characteristics were similar between all eligible survivors and those participating in the follow-up at 3 and 12 months. By 3 months, 128/262 (49%) had participated in any rehabilitation, and at 12 months 130/215 (60%). Approximately 40% were nonnative Swedish speakers, similar at both 3 and 12 months.

**Table 1.** Demographics, clinical characteristics, and outcomes.

Variable	All eligible survivors ***N*** = 299	3-month follow-up ***n*** = 262	12-month follow-up ***n*** = 215
**Demographics**			
Age, years	61 [52–68]	61 [52–68]	62 [53–69]
Female, ***n*** (%)	78 (26)	71 (27)	59 (28)
Native Swedish speaker, ***n*** (%)	N/A	152 (59)	130 (61)
Working prior,**^a^** ***n*** (%)	N/A	119 (45)	104 (48)
Education >12 years, ***n*** (%)	N/A	91 (31)	76 (35)
Frailty, CFS ≥ 5, ***n*** (%)	11 (4)	8 (3)	5 (2)
BMI (kg/m^2^)	31 [27–36]	31 [27–36]	31 [27–35]
Hypertension, ***n*** (%)	150 (51)	127 (50)	110 (52)
Diabetes mellitus,^b^ ***n*** (%)	85 (29)	72 (28)	63 (29)
Respiratory disease,^b^ ***n*** (%)	51 (17)	48 (18)	41 (19)
Cardiovascular disease,**^b^** ***n*** (%)	37 (12)	32 (12)	29 (13)
Malignant disease,**^b^** ***n*** (%)	19 (6)	14 (5)	11 (5)
Chronic kidney disease,**^b^** ***n*** (%)	8 (3)	7 (3)	5 (2)
**ICU and hospital stay**			
ICU length of stay, days	8 [5–15]	8 [5–15]	8 [5–15]
Hospital length of stay, days	26 [16–54]	27 [16–56]	28 [17–58]
SAPS3-score, mean (SD)	56 [47–65]	56 [47–65]	57 [48–66]
Prone position, ***n*** (%)	241 (81)	211 (81)	176 (82)
Invasive ventilation, ***n*** (%)	197 (66)	173 (66)	143 (67)
Time in invasive ventilation, days	4 [0–10]	4 [0–11]	5 [0–10]
**At clinical follow-up**			
Face-to-face follow-up, ***n*** (%)	N/A	173 (66)	158 (73)
Lives alone, ***n*** (%)	N/A	9 (4)	2 (1)
Returned to work,**^c^** ***n*** (%)	N/A	66 (25)	74 (35)
Participation in rehabilitation, ***n*** (%)	N/A	128 (49)	130 (60)
Ongoing rehabilitation, ***n*** (%)	N/A	77 (31)	33 (15)
Less sleep, ***n*** (%)	N/A	119 (47)	79 (37)
More sleep, ***n*** (%)	N/A	57 (22)	52 (25)
Functional outcome, GOSE	N/A	6 [5–7]	7 [6–8]

*Note*. Continuous variables are presented as median with interquartile range unless otherwise stated and categorical values with number and percentages.

^a^ Full-time or part-time.

^b^ According to the Charlson Comorbidity Index.

^c^ Returned to work full-time or part-time, only including those working before critical COVID-19.

Abbreviations: SD, standard deviation; CFS, Clinical Frailty Score; BMI, body mass index; ICU, intensive care unit; SAPS3, Simplified Acute Physiology Score 3; GOSE, Glasgow Outcome Scale Extended.

For the questionnaires, imputation was used in 14 instances at 3 months (MFIS: 3 items; HADS: 3 items; PCL-5: 8 items) and 18 instances at 12 months (MFIS: 10 items; HADS: 0 items; PCL-5: 8 items).

### Patient-Reported Fatigue

At 3 months, 115/229 (50%) reached the cutoff for fatigue, compared to 76/190 (40%) at 12 months (Table 2).

**Table 2.** Patient-Reported Symptoms at 3 and 12 months After Critical COVID-19.

Outcome	Total score 3 months median [IQR]	Above cutoff 3 months *n* (%)	Total score 12 months median [IQR]	Above cutoff 12 months *n* (%)	3 and 12 months *P* value
Fatigue	38 [22–53]	115/229 (50)	33 [16–46]	76/190 (40)	<.001*
Anxiety	5 [2–9]	75/229 (33)	4 [2–8]	54/193 (28)	.19
Depression	4 [1–9]	68/229 (30)	4 [1–7]	43/193 (22)	.17
PTSD	11 [5–26]	38/220 (17)	10 [4–22]	25/189 (13)	.09

*Note*. Fatigue was assessed by the Modified Fatigue Impact Scale, anxiety and depression was assessed by the Hospital Anxiety and Depression Scale, and PTSD by the Posttraumatic Stress Disorder Checklist updated for DSM-5.

*Indicates *P* value < .05 and considered significant. The statistical test used was the Wilcoxon Signed Rank Test.

*Abbreviations:* IQR, interquartile range; PTSD, posttraumatic stress disorder.

**Figure 2.**
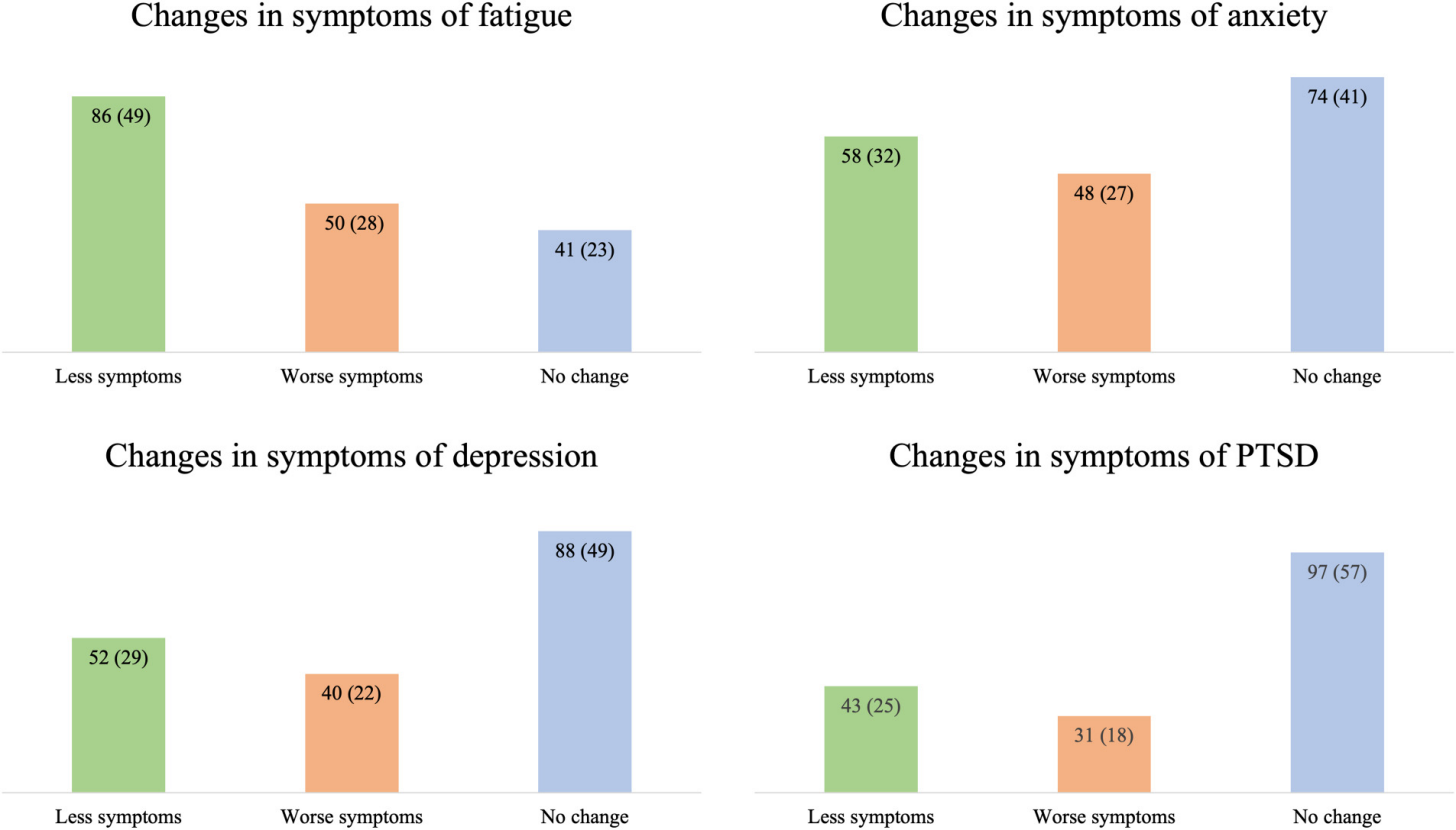
*The dynamics of fatigue and mental illness between 3 and 12 months*. The dynamics are categorized by a change in scores in relation to suggested values for a minimally important difference (MID) in the respective questionnaire, defined as: “Less symptoms” = equal to or lower than MID; “Worse symptoms” = equal to or higher than MID; “No change” = below MID, and presented with *n* (%). MID values: Modified Fatigue Impact Scale: ≥ 4. Hospital Anxiety and Depression Scale: ≥ 2; Posttraumatic Stress Disorder Checklist: ≥ 6. Number of participants with questionnaire at both 3 and 12 months included in the analyses; Modified Fatigue Impact Scale, *n* = 177; Hospital Anxiety and Depression Scale, *n* = 180; Posttraumatic Stress Disorder Checklist updated for DSM-5, *n* = 171. *Abbreviations*: PTSD, posttraumatic stress disorder.

The difference in symptoms of fatigue between 3 and 12 months was significant (median = 38 vs 33, *n* = 177, *P *< .001) and reached the threshold for being of minimal important difference (MID ≥ 4). As presented in Figure 2, 86/177 (49%) had less severe symptoms at 12 compared to 3 months. Physical fatigue had the highest impact at 3 and 12 months (Table 3) and showed the greatest improvement (mean = 2.1 vs 1.8).

**Table 3.** The Subscales of the Modified Fatigue Impact Scale at 3 and 12 Months after Critical COVID-19.

Modified Fatigue Impact Scale	3 months, mean (SD) *n* = 229	12 months, mean (SD) *n* = 190
Physical impact	2.1 (1.0)	1.8 (1.1)
Cognitive impact	1.5 (1.0)	1.4 (1.0)
Psychosocial impact	1.8 (1.2)	1.5 (1.1)

Each item on the MFIS is scored on an ordinal scale from 0 to 4 based on symptom frequency. 0 = never, 1 =rarely, 2 = sometimes, 3 = often, 4 = almost always. Abbreviations: SD, standard deviation.

### Patient-Reported Mental Illness

The proportion of participants with significant symptoms of anxiety was 75/229 (33%) at 3 months and 54/193 (28%) at 12 months (Table 2). The corresponding proportions for depression were 68/229 (30%) and 43/193 (22%) at 3 and 12 months. At 3 and 12 months, 38/220 (17%) and 25/189 (13%) reported symptoms of PTSD above cutoff. Overall, 93/226 (41%) and 67/192 (35%) scored above the cutoff for at least 1 symptom of mental illness at 3 and 12 months, respectively.

There was no significant difference in symptom severity among participants completing the questionnaires for mental illness at either point (HADS: *n* = 180; PCL-5: *n* = 171). On a group level, the change in median scores between 3 and 12 months was below the threshold for a MID for both questionnaires. At an individual level, an unchanged score (below MID) between 3 and 12 months was the most common, but some participants had either lower (less symptoms) or higher (worse symptoms) scores at 12 compared to 3 months (Figure 2).

As seen in Appendix Table A1, nonnative Swedish-speaking participants had a higher median score for fatigue and mental illness compared to native speakers at 3 and 12 months. There was a significant difference between the groups for all symptoms of mental illness at 12 months but not for fatigue.

### Risk Factors at 12 Months

The multivariable regression model showed that a worse functional outcome as assessed by the GOSE (*P *< .001) and less sleep (*P *= .008) were associated with anxiety at 12 months (Table 4). A worse functional outcome by the GOSE was the only variable significantly associated with depression (*P *< .001). Due to few events (n = 25 above the cutoff), only 2 variables were included in the multivariable regression model for PTSD, of which both diabetes mellitus (*P *= .002) and less sleep (*P *= .001) were significant. In the multivariable regression model for fatigue, preadmission cardiovascular disease (*P *= .009), worse functional outcome as assessed by the GOSE (*P *< .001) and less sleep (*P *= .006) were associated with fatigue at 12 months. Univariable analyses and the selection for the multivariable analyses are shown in Appendix Tables B1–B4.

**Table 4.** Multivariable Logistic Regression of Factors Associated with Long-Lasting Symptoms after Critical COVID-19.

Variable	OR [95% CI]	mOR [95% CI]	*P* value
Anxiety			
Male sex	0.51 [0.25–1.03]	0.67 [0.28–1.16]	.37
SAPS3-score	1.03 [1.01–1.06]	1.03 [0.99–1.07]	.07
Hypertension	1.66 [0.84–3.26]	1.16 [0.51–2.65]	.73
Functional outcome (GOSE)	0.35 [0.24–0.52]	0.40 [0.26–0.61]	<.001*
Less sleep	3.73 [1.89–7.35]	2.94 [1.26–6.85]	.008*
			HL-test: .75
Depression			
Age, years	0.97 [0.94–1.00]	0.97 [0.94–1.00]	.08
Diabetes mellitus^a^	1.72 [0.83–3.55]	1.34 [0.55–3.24]	.52
Functional outcome (GOSE)	0.39 [0.27–0.57]	0.43 [0.29–0.63]	<.001*
Less sleep	3.52 [1.70–7.28]	1.86 [0.82–4.25]	.14
			HL-test: .52
PTSD			
Diabetes mellitus^a^	4.58 [1.89–11.07]	4.35 [1.73–10.93]	.002*
Less sleep	4.14 [2.21–7.75]	5.22 [1.91–14.25]	.001*
			HL-test: .28
Fatigue			
Prone position	1.84 [0.8–4.11]	2.80 [0.97–8.10]	.06
Hypertension	2.28 [1.23–4.22]	2.10 [0.89–4.8]	.09
Diabetes mellitus^a^	2.01 [1.06–3.82]	0.95 [0.38–2.33]	.91
Cardiovascular disease^a^	3.95 [1.54–10.16]	4.64 [1.47–14.70]	.009*
Functional outcome (GOSE)	0.34 [0.23–0.50]	0.38 [0.24–0.59]	<.001*
Less sleep	4.14 [2.21–7.75]	2.94 [1.37–6.32]	.006*
More sleep	2.51 [1.27–4.99]	1.68 [0.72–3.92]	.23
			HL-test: .21

^a^ From the Charlson comorbidity index.

*Indicates *P* value < .05 and considered significant.

Abbreviations: CI, confidence interval; OR, odds ratio; mOR, multivariable odds ratio; HL, Hosmer & Lemeshow test; SAPS3, Simplified Acute Physiology Score 3; GOSE, Glasgow Outcome Scale Extended.

The Hosmer–Lemeshow test was used to evaluate the model calibration.

## Discussion

This study demonstrates that 12 months after admission to intensive care for critical COVID-19, 40% report significant fatigue and approximately one-third of the participants still experience significant symptoms of mental illness. A significant improvement over time was seen for fatigue but not for symptoms of mental illness. A worse functional outcome as assessed by the GOSE and less sleep compared to before COVID-19 were risk factors for reporting long-lasting symptoms.

We report that fatigue was the most common symptom among all investigated symptoms and substantially more common than in the general Swedish population.^34^ This may illustrate the long-term impact of respiratory failure and intensive care in general, and is in line with previous studies of COVID-19, with fatigue being the most common lasting symptom.^1^ The proportion of fatigue reported here was lower than reported in similar observational studies on survivors of ARDS and critical COVID-19 requiring intensive care.^9,10^

An improvement in fatigue during the first year has previously been reported for survivors of ARDS,^9^ but has, to our knowledge, not previously been shown in survivors of critical COVID-19. The improvement in fatigue seems to be related to previously reported improvements in physical aspects of health over time (Didriksson et al, unpublished) as physical fatigue showed the most improvement. However, physical fatigue still had the largest impact at both points. Although a significant improvement in fatigue was seen during the first year, a substantial proportion of the participants continued to report significant fatigue at 12 months. This is important as fatigue has been shown to have a considerable impact on a person's daily life in other populations—such as cancer and cardiac arrest—and is associated with difficulties in returning to work and decreased societal participation.^35,36^

In this study symptoms of mental illness were mostly unchanged on a group level during the first year, which is in line with previous studies of post-ARDS and critical illness.^7,37,38^ At an individual level, however, this was not the case. Overall, our cohort had higher levels of anxiety and depression than the general Swedish population.^39^ Furthermore, our cohort also reported a higher frequency of symptoms of mental illness than a similar population in the Netherlands, where 26%, compared to 35% in our study, reported symptoms of mental illness 12 months after critical COVID-19.^10^ One explanation for this result could be the use of different instruments, or in the amount of missing data, with 46% in the Dutch study versus 29% in the current study. Another potential explanation could be differences in the healthcare provided. Still, since both studies lack information on interventions and support for mental illness during the first year, this could not be investigated.

The results that the illness severity during ICU care had no impact on lasting symptoms is slightly unexpected but is in line with previous reports from survivors of ARDS.^7,9,37,40^ These results are interesting as they may implicate a need for post-ICU fatigue and mental illness screening regardless of acute disease severity. Another surprising finding was that sex was not associated with fatigue or mental illness, which is in contrast with previous findings where female sex was a risk factor for both PACS and PICS.^41,42^ Since most of our participants were males, some effects might have been lost.

Our finding that functional outcome as assessed by the GOSE is associated with fatigue and mental illness at long-term follow-up after critical COVID-19 was expected. We speculate that functional outcome is interrelated with all investigated symptoms, as a loss of function, for example, not returning to work or the inability to perform a previous hobby, could lead to symptoms of depression. On the other hand, fatigue might lead to a worse functional outcome as a lack of energy may decrease the ability to return to work or reengage with hobbies. These results indicate that survivors with a worse functional outcome may need extra support and rehabilitation.

Though 2 distinct conditions, there is an association between feelings of fatigue and depression. The symptoms are often closely interlinked and might reinforce one another. An example of their close association is that a part of depressive symptomology in DSM-5 is fatigue.^43^ Healthcare professionals who encounter patients with a chief complaint of feeling fatigued should also have depression as a differential diagnosis in mind. While depression and fatigue are closely associated, fatigue is also common without depression in COVID-19 and ARDS, indicating that there are other reasons for fatigue and the need to screen for these symptoms separately.^1,9^

Less sleep was also associated with fatigue and all symptoms of mental illness, except for depression. As symptoms of mental illness are commonly linked with sleeping disorders, this finding was not surprising.^44–46^ Although sleeping disorders are not uncommon in the general population,^47^ the “less sleep” reported here relates to a change compared to previous habits, and our results indicate new problems with less sleep in 37% of participants at 12 months. Good sleep hygiene and other treatments may improve sleep quality and quantity, but whether this could affect symptoms of fatigue and mental illness is unclear and should be further investigated in forthcoming studies.

The observation time of up to 1 year in the present study adds to the growing body of evidence regarding the long-term effects of critical COVID-19. The results could have implications for the multidisciplinary management of survivors of critical COVID-19, and possibly for other post-ICU care. Our study highlights which symptoms need to be screened for in health and care settings, and particularly in patients recovering from critical COVID-19 or non-COVID-19 critical respiratory failure. Although not included in our main analyses, our results indicate that the nonnative Swedish-speaking participants have more pronounced symptoms than the native-speaking participants. Future studies need to explore whether this is related to the different impacts of critical COVID-19 or other factors.

The strengths of this study include the fact that participants were enrolled at the time of admission to the ICU, that clinical data were prospectively collected in a structured database, and that long-term symptoms were assessed on 2 occasions. This generated an opportunity to explore potential changes over time and to identify associated factors rendering lasting symptoms. In addition, the multicenter design and the study population's size increase our results’ generalisability.^48^

Limitations of the study include some loss-to-follow-up, although the amount of missing data (13% at 3 months and 29% at 12 months) was still acceptable.^48^ Another limitation is the difficulty in comparing the results with other studies due to the heterogeneity in the field, especially regarding the use of different instruments to measure patient-reported outcomes.^48^ One specific challenge is that although the HADS is included in the core outcome measures for Clinical Research in Acute Respiratory Failure, the same recommendation includes the Impact of Event Scale (IES) rather than the PCL-5 used here.^20^ We chose the PCL-5 as it fully aligns with the DSM-5 criteria, which the IES does not do.^49^ We cannot rule out that our results would have differed if we had used the IES. Furthermore, the reference values for cutoffs and MIDs are mostly not based on populations with COVID-19 or ARDS/critical illness, which should be considered. An important limitation is that the severity of symptoms cannot be put into a context with a baseline score before COVID-19. It is thus unknown if symptoms of fatigue and mental illness fatigue were on the same level as the Swedish general population before critical COVID-19, and the study cannot differentiate between preexisting, new or worse symptoms. Finally, the patient-reported symptoms do not equal a medical diagnosis. To what extent the symptoms represent a clinical diagnosis of mental illness needs to be further evaluated.^50^

## Conclusions

Our results emphasize the high prevalence of symptoms of fatigue and mental illness in survivors of critical COVID-19. While some individuals improve over time, symptoms remain in a substantial proportion of the population for at least 12 months. Screening for symptoms of fatigue and mental illness is crucial to provide appropriate support and treatment to improve patient-related outcomes. Based on the results, screening should be performed and repeated during the first year.

## Appendix A

Native versus nonnative Swedish speakers, patient-reported symptoms.

**Table A1.** Native Versus Nonnative Swedish Speakers, Patient-Reported Symptoms at 3 and 12 Months.

Outcome	3 months Total score, median [IQR]		*P* value	12 months Total score, median [IQR]		*P* value
	Native	Nonnative		Native	Nonnative	
Fatigue	37 [24–53]	40 [21–51]	.68	31 [14–44]	37 [20–52]	.08
Anxiety	4 [1–8]	6 [3–11]	.002*	3 [1–7]	6 [3–10]	<.001*
Depression	3 [1–8]	6 [1–9]	.13	3 [1–6]	5 [2–9]	.006*
PTSD	9 [4–22]	16 [7–31]	.009*	7 [3–17]	15 [4–31]	.007*

Fatigue was assessed by the Modified Fatigue Impact Scale, anxiety and depression was assessed by the Hospital Anxiety and Depression Scale, and PTSD was assessed by the Posttraumatic Stress Disorder Checklist updated for DSM-5. “Native” and “nonnative” are in reference to the Swedish language.

*Indicates a *P* < .05 and considered significant. The test used was the independent samples Mann–Whitney *U* test.

Abbreviations: IQR, interquartile range; PTSD, posttraumatic stress disorder.

## Appendix B

Univariable analyses and the selection of variables to the multivariable analyses.

**Table B1.** Univariable Analysis of Variables Potentially Associated with Symptoms of Fatigue at 12 Months.

Univariable analysis—Fatigue		
Variable	*P* value	*OR* [95% CI]
Age	.56	1.007 [0.98–1.03]
Sex	.55	0.82 [0.42–1.58]
BMI	.36	1.02 [0.97–1.08]
ICU LOS	.71	0.99 [0.98–1.02]
SAPS3-score	.40	1.01 [0.99–1.04]
Prone position	.14^b^*	1.84 [0.8–4.11]
Invasive ventilation	.58	0.83 [0.45–1.57]
Hypertension	.009^b^*	2.28 [1.23–4.22]
Diabetes mellitus^a^	.03^b^*	2.01 [1.06–3.82]
Cardiovascular disease^a^	.004^b^*	3.95 [1.54–10.16]
Chronic pulmonary disease^a^	.78	1.11 [0.53–2.34]
Rehabilitation	.25	0.70 [0.38–1.29]
Functional outcome (GOSE)	<.001^b^*	0.34 [0.23–0.50]
Less sleep	<.001^b^*	4.14 [2.21–7.75]
More sleep	.008^b^*	2.51 [1.27–4.99]

^a^ From the Charlson comorbidity index.

^b^ Signifies variables included in the multivariable model.

*Indicates a *P* value < .25 and considered significant.

Abbreviations: CI, confidence interval; OR, odds ratio; BMI, body mass index; ICU, intensive care unit; LOS, length of stay; SAPS3, Simplified Acute Physiology Score 3; GOSE, Glasgow Outcome Scale Extended.

**Table B2.** Univariable Analysis of Variables Potentially Associated with Symptoms of Anxiety at 12 Months.

Univariable analysis—Anxiety		
Variable	*P* value	OR [95% CI]
Age	.22*	0.98 [0.96–1.01]
Sex	.06^b^*	0.51 [0.25–1.03]
BMI	.23*	1.04 [0.98–1.09]
ICU LOS	.76	1.003 [0.98–1.03]
SAPS3-score	.02^b^*	1.03 [1.01–1.06]
Prone position	.43	1.45 [0.56–3.66]
Invasive ventilation	.35	1.40 [0.69–2.84]
Hypertension	.14^b^*	1.66 [0.84–3.26]
Diabetes mellitus^a^	.62	1.19 [0.60–2.35]
Cardiovascular disease^a^	.92	0.95 [0.37–2.46]
Chronic pulmonary disease^a^	.16*	1.82 [0.79–4.18]
Rehabilitation	.59	1.21 [0.61–2.37]
Functional outcome (GOSE)	<.001^b^*	0.35 [0.24–0.52]
Less sleep	<.001^b^*	3.73 [1.89–7.35]
More sleep	.15*	1.67 [0.83–3.35]

^a^ From the Charlson comorbidity index.

^b^ Signifies variables included in the multivariable model.

*Indicates a *P* value < .25 and considered significant.

Abbreviations: CI, confidence interval; OR, odds ratio; BMI, body mass index; ICU, intensive care unit; LOS, length of stay; SAPS3, Simplified Acute Physiology Score 3; GOSE, Glasgow Outcome Scale Extended.

**Table B3.** Univariable Analysis of Variables Potentially Associated with Symptoms of Depression at 12 Months.

Univariable analysis—Depression		
Variable	*P* value	OR [95% CI]
Age	.06^b^*	0.97 [0.94–1.00]
Sex	.83	0.92 [0.42–1.99]
BMI	.76	1.009 [0.95–1.07]
ICU LOS	.83	1.002 [0.98–1.03]
SAPS3-score	.28	1.02 [0.99–1.05]
Prone position	.88	1.08 [0.43–2.70]
Invasive ventilation	.85	1.07 [0.51–2.24]
Hypertension	.35	1.41 [0.69–2.88]
Diabetes mellitus^a^	.14^b^*	1.72 [0.83–3.55]
Cardiovascular disease^a^	.82	1.13 [0.41–3.09]
Chronic pulmonary disease^a^	.33	1.53 [0.66–3.57]
Rehabilitation	.72	0.88 [0.43–1.79]
Functional outcome (GOSE)	<.001^b^*	0.39 [0.27–0.57]
Less sleep	<.001^b^*	3.52 [1.70–7.28]
More sleep	.26	1.53 [0.73–3.21]

^a^ From the Charlson comorbidity index.

^b^ Signifies variables included in the multivariable model.

*Indicates a *P* value < .25 and considered significant.

Abbreviations: CI, confidence interval; OR, odds ratio; BMI, body mass index; ICU, intensive care unit; LOS, length of stay; SAPS3, Simplified Acute Physiology Score 3; GOSE, Glasgow Outcome Scale Extended.

**Table B4.** Univariable Analysis of Variables Potentially Associated with Symptoms of Posttraumatic Stress Disorder at 12 Months.

Univariable analysis—PTSD		
Variable	*P* value	OR [95% CI]
Age	.68	0.99 [0.96–1.03]
Sex	.25	0.59 [0.24–1.14]
BMI	.05*	1.07 [1.00–1.15]
ICU LOS	.42	0.99 [0.95–1.02]
SAPS3-score	.02*	1.04 [1.01–1.08]
Prone position	.68	0.80 [0.27–2.33]
Invasive ventilation	.33	0.65 [0.27–1.55]
Hypertension	.09*	2.24 [0.88–5.69]
Diabetes mellitus^a^	<.001^b^*	4.58 [1.89–11.07]
Cardiovascular disease^a^	.11*	2.32 [0.82–6.56]
Chronic pulmonary disease^a^	.42	0.59 [0.16–2.12]
Rehabilitation	.54	0.77 [0.33–1.81]
Functional outcome (GOSE)	<.001*	0.35 [0.24–0.53]
Less sleep	<.001^b^*	5.47 [2.06–14.52]
More sleep	.73	0.84 [0.31–2.25]

^a^ From the Charlson comorbidity index.

^b^ Signifies variables included in the multivariable model.

*Indicates a *P* value < .25 and considered significant.

Abbreviations: CI, confidence interval; OR, odds ratio; BMI, body mass index; ICU, intensive care unit; LOS, length of stay; SAPS3, Simplified Acute Physiology Score 3; GOSE, Glasgow Outcome Scale Extended.
